# Syntaxin-4, a key exocytosis mediating protein, shows heterogeneous expression in insulin-positive cells of human donors with new-onset and longer duration of type 1 diabetes: comparison with non-diabetic autoantibody-positive and -negative donors

**DOI:** 10.1007/s10735-026-10901-4

**Published:** 2026-07-30

**Authors:** Joshua Potman, Kevin Xueying Sun, Kaaj Pala, Lars Krogvold, Knut Dahl-Jørgensen, Shiva Reddy

**Affiliations:** 1 Department of Molecular Medicine and Pathology, Faculty of Medical and Health Sciences, University of Auckland, Auckland, Auckland, 1023 New Zealand; 2 Division of Paediatric and Adolescent Medicine, Oslo University Hospital, Oslo, Norway; 3 Faculty of Dentistry, University of Oslo, Oslo, Norway; 4 Faculty of Medicine, University of Oslo, Oslo, Norway; 5 Oslo University Hospital, Oslo, Norway; 6 Department of Molecular Medicine and Pathology, Faculty of Medical and Health Sciences, University of Auckland, Auckland, New Zealand

**Keywords:** Human type 1 diabetes, Insulin secretion, Islets, Syntaxin-4, Immunohistochemistry

## Abstract

Syntaxin-4, a beta cell plasma membrane-anchored protein, attaches to beta cell granules with two spatially-located nearby proteins, facilitating the fusion of beta cell-docked granules to the plasma membrane, followed by exocytotic release of insulin and remaining granule contents. During type 1 diabetes (T1D) and its preceding phase, it is unclear whether syntaxin-4 levels decline in residual beta cells, thereby contributing to impaired insulin release. The level of syntaxin-4 in insulin-positive regions of islets was determined following immunofluorescence staining of human pancreatic sections, with co-immunofluorescence staining for insulin and for glucagon by immunoperoxidase. Random islets from non-diabetic autoantibody-negative (Group 1; *n* = 7), non-diabetic autoantibody-positive (Group 2; *n* = 6), new-onset T1D (Group 3; *n* = 4) and long-term T1D (Group 4; *n* = 10) donors were quantified for syntaxin-4 levels. In groups 1 and 2, inter-islet staining intensities for syntaxin-4, although variable across individual donors, were similar overall between the groups, with lower intensities in group 3. In group 4, 3 donors with 0.58–1.5 years of T1D showed mean intensities comparable to groups 1 and 2, whereas donors with 4–7 years of T1D showed declining intensities. In general, the average syntaxin-4 intensities in the two diabetic groups were lower than those in the non-diabetic groups. The expression of syntaxin-4 in insulin-positive islet cells showed marked differences in intensity among most diabetic donors and autoantibody-positive and -negative non-diabetic donors. In some long-term diabetic donors, syntaxin-4 was also present in insulin- and glucagon-negative cells. During protracted T1D, reduced syntaxin-4 in islets may impair insulin release.

**Supplementary Information:**

The online version contains supplementary material available at 10.1007/s10735-026-10901-4.

## Introduction

In the human pancreas, most insulin-positive beta cells reside within at least a million randomly-located islets, with closely juxtaposed glucagon, somatostatin, pancreatic polypeptide and ghrelin endocrine cells (Cabrera et al. 2006; Kim et al. 2009). In non-diabetic individuals, single and aggregated insulin cells are also present as small clusters with or without glucagon cells (Drotar et al. 2025; Lehrstrand et al. 2024). In some subjects destined to develop type 1 diabetes (T1D), beta cells experience variable rates of declining insulin secretion in response to glucose prior to the onset of clinical diagnosis (Evans-Molina et al. 2018, 2025; Sosenko et al. 2013; Srikanta et al. 1984). During T1D, beta cell loss or its functional impairment can proceed at different rates (Atkinson et al. 2011, 2014; Atkinson and Eisenbarth 2001; Atkinson and Mirmira 2023; Roep et al. 2021). Immunohistochemical (IHC) analyses of rare pancreatic sections from deceased donors with variable duration of disease demonstrate asynchronous destruction of inter- and intra-islet beta cells and persistence of some beta cells over a prolonged period (Campbell-Thompson et al. 2016; Reddy et al. 2015). However, whether the final exocytotic process from plasma membrane-docked beta cell granules is impaired in residual beta cells during clinical T1D and in the preceding period remains unclear.

In diabetic and non-diabetic subjects, insulin and C-peptide are co-secreted with islet amyloid polypeptide, some intact proinsulin, and other contents from beta-cell vesicular stores. In many subjects with long-standing T1D, varying levels of serum and urinary C-peptide are detectable, indicating preservation of partial or intact insulin secretory capacity (Davis et al. 2015; Herold et al. 2015; Madsbad et al. 1978; McGee et al. 2014; Oram et al. 2014, 2015; Palmer et al. 2004; Wang et al. 2012; Yu et al. 2019). These results agree with IHC findings showing varying numbers of insulin-positive islets in some donors with new-onset disease and during prolonged disease (Keenan et al. 2010; Morgan and Richardson 2018; Pala et al. 2025; Reddy et al. 2015).

The molecular steps which promote docking, priming and the ultimate granule-plasma membrane fusion involve the obligatory formation of a complex of three mandatory protein components called SNARE (soluble N-ethylmaleimide-sensitive factor attachment protein receptor) proteins (Aslamy and Thurmond 2017; Ma et al. 2004). Immediately prior to exocytosis, the SNARE proteins form a trimeric complex consisting of one vesicle-(v-) SNARE (vesicle-associated membrane protein (VAMP2; molecular weight 18 kDa) and two plasma membrane-anchored t-SNARE proteins, termed syntaxin-4 (molecular weight: 35 kDa) or syntaxin-1 (molecular weight 33 kDa) and the synaptosome-associated membrane proteins, SNAP-23 or SNAP-25 (molecular weight 23 kDa or 25 kDa, respectively) (Xie et al. 2015). Granule-plasma membrane attachment via the above SNARE proteins is regulated by intracellular calcium and accessory-binding proteins, such as Sec1/Munc 18-like (SM), Munc 13 and DOC2 (double C2-domain containing proteins) (Aslamy and Thurmond 2017).

Within beta cells, syntaxin-4 has been proposed to be obligatory for mediating the final exocytotic process, as reduced syntaxin-4 levels impair biphasic glucose-stimulated insulin secretion (Aslamy and Thurmond 2017; Xie et al. 2015). However, whether human beta cells express lower levels of syntaxin-4 in situ before clinical diagnosis or after the onset of T1D remains to be determined. In cultured human islets, the depletion of syntaxin-4 suppresses the first and second phases of insulin release, whereas its upregulation in islets from type 2 diabetic donors augments insulin secretion (Oh et al. 2014; Xie et al. 2015). Whether these in vitro findings mimic in vivo pathological secretory processes remain to be clarified. In the non-obese diabetic (NOD) mouse model of T1D, a reduced expression of syntaxin-4 is observed (Oh et al. 2021). In the same studies, in vivo up-regulation of syntaxin-4 resulted in 73% of mice exhibiting normoglycaemia by 25 weeks of age, compared with < 20% in control NOD mice.

Despite studies supporting an essential role for syntaxin-4 in the final exocytotic process, its expression levels in beta cells from donors with and without T1D remain poorly characterised. To date, available IHC data have been derived from the analysis of a limited number of islets from only three human paediatric donors (Oh et al. 2021). Given the pronounced intra- and inter-islet cellular heterogeneity in T1D and the confounding impact of age on disease progression, a more comprehensive evaluation of syntaxin-4 levels across a larger cohort of donors and analysis of a greater number of insulin-positive islets are warranted. Furthermore, syntaxin-4 expression has yet to be investigated in long-standing T1D cases or in autoantibody-positive, non-diabetic individuals exhibiting beta-cell dysfunction who may be at high risk of progressing to clinical disease.

To address some of these major knowledge gaps, we aimed to systematically evaluate syntaxin-4 expression in a larger number of islets from an expanded cohort of human donors. We developed and validated a robust triple-label IHC protocol combining syntaxin-4 with insulin and glucagon staining in human pancreatic sections. This optimised approach enabled us to precisely assess cellular identity and quantify syntaxin-4 levels within beta-cell areas of randomly selected islets. To overcome certain limitations of prior reported studies, we extended our analysis to well-characterised age-matched cohorts, including non-diabetic controls with or without islet cell autoantibodies, as well as donors with newly diagnosed or long-standing T1D (Krogvold et al. 2014; Pugliese et al. 2014).

## Materials and methods

### Pancreatic samples and study groups

Formalin-fixed, paraffin-embedded sections(5 μm) were prepared from pancreatic tail region biopsies obtained from 4 living individuals with newly diagnosed type 1 diabetes (from the Diabetes Virus Detection [DiViD] Study) and 23 cadaveric donors (supplied by the Network for Pancreatic Organ Donors with Diabetes [nPOD]) (Krogvold at al. 2014, Pugliese et al. 2014). For additional validation of our IHC protocol, sections were tested from archived formalin-fixed rat pancreas, human palatine tonsil, and Bouin's-fixed mouse pancreas. Human tonsil sections were used as negative controls, since syntaxin-4 has been reported to be undetectable by immunohistochemistry (Human Protein Atlas). Study groups and donor demographics are summarized in Tables 1 and 2, respectively.

**Table 1:** Summary of main study groups, including sex and age distribution

Study group	Sex (no. female/no. male)	Age (years)	
		Mean ± SD	Median (range)
Group 1 (non-diabetic AAb-negative)	2/5	26.66±8.57	24.5 (19–44)
Group 2 (non-diabetic AAb-positive)	2/4	28.66±8.56	27 (17.65–40.3)
Group 3 (new-onset diabetes)	2/2	28.50±4.80	28(24–31)
Group 4 (long-term diabetes)	4/6	27.96±7.11	26.52(20.7–44)

AAb, autoantibody; no., number; SD, standard deviation

**Table 2:** Demographic information and case characteristics

Case ID (RRID for nPOD cases)	AAb positivity (Type)	Duration of T1D	Age at death/biopsy (years)	Sex, ethnicity, BMI	C-peptide (ng/mL)	Cause of death (relevant history and pancreatic disorders – nPOD cases only)
*Group 1: Non-diabetic AAb-negative cases (nPOD)*						
6289 (SAMN15879343)	–	–	19	M, Af-Am, 38.3	8.05	Head trauma
6234 (SAMN15879290)	–	–	20	F, white, 25.6	6.89	Head trauma
6160 (SAMN15879216)	–	–	22.1	M, white, 23.9	0.4	Head trauma
6178 (SAMN15879234)	–	–	24.5	F, white, 27.5	4.55	Anoxia
6055 (SAMN15879112)	–	–	27	M, white, 22.7	0.59	Anoxia
6048 (SAMN15879105)	–	–	30	M, white, 20.6	17.91	Cerebrovascular/stroke
6369 (SAMN15879422)	–	–	44	M, white, 18.8	6.42	Cerebrovascular/stroke
*Group 2: Non-diabetic AAb-positive cases (nPOD)*						
6424 (SAMN15879477)	2AAb (GADA, IAA)	–	17.65	M, white, 51.4	6.97	Head trauma
6267 (SAMN15879321)	2AAb (GADA, IA-2A)	–	23	F, white, 23.5	16.59	Anoxia
6301 (SAMN15879355)	1AAb (GADA)	–	26	M, Af-Am, 32.08	3.92	Head trauma
6310 (SAMN15879364)	1AAb (GADA)	–	28	F, Hispanic, 22.4	10.54	Anoxia; low grade insulitis
6167 (SAMN15879223)	2AAb (IA-2 A, ZnT8A)	–	37	M, white, 26.3	5.43	Head trauma
6158 (SAMN15879214)	2AAb (GADA, IAA)	–	40.3	M, white, 29.7	0.51	Head trauma
*Group 3: Newly-diagnosed diabetic cases (DiViD)*						
Case 1	4AAb (GADA, IA-2A, IAA, ZnT8A)	4 weeks	25	F, white	1.11	Live donor
Case 2	3AAb (GADA, IA-2A, ZnT8A)	3 weeks	24	M, white	1.05	Live donor
Case 3	3AAb (GADA, IA-2A, ZnT8A)	9 weeks	34	F, white	2.40	Live donor
Case 4	3AAb (GADA, IA-2A, IAA)	5 weeks	31	M, white	NA	Live donor
*Group 4: Long-term diabetic cases (nPOD)*						
6551 (SAMN25652262)	4AAb (GADA, IA-2A, IAA, ZnT8A)	0.58 year	20.7	M, white, 23.1	0.11	Anoxia
6593 (SAMN38117312)	4AAb (GADA, IA-2A, IAA, ZnT8A)	1 year	26.64	M, white, 29.5	0.02	Anoxia
6469 (SMAN15879522)	1A-Ab (GADA)	1.5 years	27.06	F, white, 26.9	0.66	Anoxia
6211 (SAMN15879267)	4AAb (GADA, IA-2A, IAA, ZnT8A)	4 years	24	F, Af-Am, 24.4	< 0.05	Anoxia; Very mild chronic inflammation within interstitial fibrosis and mononuclear infiltration (2 foci)
6088 (SAMN15879145)	4AAb (GADA, IA-2A, IAA, ZnT8A)	5 years	31.2	M, white, 27	< 0.05	Head trauma; mild, chronic pancreatitis, insulitis
6070 (SAMN15879127)	2AAb (IA-2A, IAA)	7 years	22.6	F, white, 21.6	< 0.05	Anoxia
6245 (SAMN15879301)	2AAb (GADA, IA-2A)	7 years	22	M, white, 23.2	< 0.05	Head trauma; very mild chronic inflammation, insulitis- sparse, multiple hospitalizations with DKA
6045 (SAMN15879102)	2AAb (IAA, ZnT8A)	8 years	26.4	M, white, 23.1	< 0.05	Head trauma
6262 (SAMN15879316)	3AAb (GADA, IA-2A, IAA)	8 years	44	M, Af-Am, 21.5	< 0.05	Anoxia; very mild chronic inflammation
6220 (SAMN15879276)	2AAb (GADA, IAA)	11 years	35	F, white, 27.4	< 0.05	Anoxia

AAb, autoantibody; Af-Am, African American; BMI, body mass index (kg/m2); DKA, diabetic ketoacidosis; EADB, Exeter Archival Diabetes Biobank; F, female; GADA, anti-glutamic acid decarboxylase; IA-2A, anti-insulinoma-associated antigen; IAA, insulin autoantibodies; M, male; NA, not available; T1D, type 1 diabetes; ZnT8A, anti-zinc transporter 8.

Cases 1–4 were from the DiViD Study where sections were prepared from pancreatic biopsies from living donors with recent onset T1D. All nPOD sections, were from cadaveric pancreas.

Case 6424 had a BMI 51.1, an outlier from the rest of the cases selected, but included due to his non-diabetic autoantibody positive status. Each nPOD case has a unique Research Resource Identifier (RRID) beginning with “SAMN”.

## Triple-label IHC protocol

Antibodies, key reagents, and incubation protocols are summarized in Table 3. Syntaxin-4 was localised using a rabbit polyclonal antibody raised against a 2–23 amino acid fragment of rat syntaxin-4, which shares 17 amino acid sequence identity with the human homologue. The molecular weight of human syntaxin-4 has been reported as 34.18 kDa (UniProt Database) and is close to the value of 34–35 kDa reported by the supplier and by others (Aslamy and Thurmond 2017). Syntaxin-4 antibody was used at a working dilution (2.67 µg/ml IgG). The combined immunofluorescence (IF) and immunoperoxidase (IPO) protocol and subsequent haematoxylin counterstaining procedure have been previously reported and are briefly described below (Pala et al. 2025; Reddy et al. 2018, 2021). Briefly, syntaxin-4 intensity in pancreatic islets was assessed via IF, followed by co-labelling for insulin (IF) and glucagon (IPO).

**Table 3:** List of antibodies and reagents for immunohistochemistry in the study

Antibodies/Reagents	Supplier	Catalogue number	Working Dilution	Diluent	Incubation time/temperature
*Primary antibodies*					
Rabbit anti-syntaxin-4	Alomone	ANR-004	1:150	CST diluent	18 h, 4 °C
Guinea pig anti-insulin	Dako	AO564	1:600	CST diluent	1.5 h, 37 °C
Mouse anti-glucagon	Sigma-Aldrich	SAB4200685	1:600	CST diluent	1.5 h, 37 °C
Mouse anti-human CD45	Abcam	ab781	1:50	CST diluent	18 h, 4 °C
*Secondary antibodies*					
Donkey anti-rabbit IgG (H + L) Alexa 568	Invitrogen	A10042	1:600	0.2% PBST	1 h, 37 °C
Donkey anti-guinea pig IgG Alexa 488	Jackson ImmunoResearch	706-545-148	1:300	0.2% PBST	1 h, 37 °C
Goat anti-mouse IgG (Fab specific) peroxidase	Sigma Aldrich	A2304	1:200	0.2% PBST	1 h, 37 °C
Horse anti**-**mouse IgG (H + L) Dylight 488	Vector	Dl-2488	1:200	0.2% PBST	1 h, 37 °C

Abcam, Abcam (Cambridge, UK); Alomone, Alomone Labs Ltd (Jerusalem, Israel); CST, Cell Signaling Technology; Dako, Dako (Glostrup, Denmark); Invitrogen, Invitrogen (Thermo Fisher Scientific), Eugene, OR, USA; Jackson ImmunoResearch, Jackson ImmunoResearch (West Grove, PA, USA); 0.2% PBST, 0.2% volume/volume Tween20 in phosphate-buffered saline; Sigma-Aldrich, Sigma-Aldrich (Darmstadt, Germany); Vector, Vector Laboratories (Burlingame, CA, USA)

Guinea pig anti-insulin and mouse anti-glucagon antibodies have been validated for immunohistochemistry in this laboratory. Negative controls for syntaxin-4 immunostaining were performed by substituting the primary antibody with either diluent or normal rabbit IgG in the immunofluorescence procedure. Application of immunofluorescence staining for syntaxin-4 to human tonsil sections following fixation of tonsillar tissue with formalin also acted as an additional negative control. Secondary antibodies linked to Alexa dyes and peroxidase were employed in combined immunohistochemical protocol for syntaxin-4, insulin and glucagon (human, mouse and rat pancreas) and horse anti-mouse IgG Dylight 488 for staining CD45 cells (human tonsil sections only)

Sections were deparaffinized, rehydrated, and subjected to heat-induced antigen retrieval in citrate buffer (pH 6) containing 0.05% (v/v) Tween-20. Slides were then permeabilised in 0.4% (v/v) Triton-X-100 in phosphate-buffered saline (PBS) on ice for 30 min, and endogenous peroxidase activity was blocked using 3% (v/v) hydrogen peroxide in PBS for 15 min. After blocking with 10% (v/v) normal donkey serum in low protein blocking solution (eBioscience, San Diego, CA, USA) for 1 h at 37 °C, sections were incubated with rabbit anti-syntaxin-4 for 18 h at 4 °C. Subsequently, sections were incubated with donkey anti-rabbit IgG-Alexa 568 (1:600) in 0.2% (v/v) Tween-20/PBS, followed by a 1.5-h incubation with a mixture of guinea pig anti-insulin and mouse anti-glucagon. Sections were then incubated with a mixture of donkey anti-guinea pig IgG-Alexa 488 and goat anti-mouse IgG-HRP for 1 h. Glucagon-positive cells were visualised using SignalStain diaminobenzidine (DAB) substrate (Cell Signaling), monitored microscopically, and the reaction was terminated with distilled water. Slides were prepared for microscopy, left in the dark overnight at room temperature prior to microscopic examination and imaging.

For negative controls, anti-syntaxin-4 antibody was replaced with either antibody diluent or normal rabbit IgG at an equivalent concentration, followed by addition of the corresponding secondary antibody conjugate and application of identical detection and co-labelling procedures for insulin and glucagon as above. To further validate antibody specificity, sections of human palatine tonsil were employed as negative controls by co-staining for syntaxin-4 and CD45, using a mouse anti-human CD45 antibody detected via horse anti-mouse secondary antibody conjugated with Dylight488. Pancreatic sections from rat and mouse were also immunostained for syntaxin-4 by IF, followed by staining for insulin and glucagon as above.

Following acquisition of combined immunostained images from IF and IPO of almost all the islets in each human pancreatic section, slides were counterstained with haematoxylin to reveal nuclei (blue). For haematoxylin counterstaining, sections were immersed in Gill’s haematoxylin for 4 min, differentiated in running tap water and dipped in 1% (v/v) lithium carbonate for 2 min. They were washed in water, air-dried, mounted for microscopic examination. Selective islets previously imaged by IF and IPO were then identified and re-imaged by light microscopy to reveal nuclei in corresponding islets, islet boundary and surrounding exocrine regions.

## Image acquisition

Prior to haematoxylin staining, sections stained by IF and IPO were examined with a Nikon Eclipse-epifluorescence-bright field microscope, and digital images were recorded in TIFF format. For syntaxin-4 IF staining in islets, all images from different donor sections were captured using identical image acquisition settings (gain and exposure). Sections from non-diabetic donors were analyzed in a blinded manner, whereas those from diabetic cohorts could not be blinded due to their known T1D status. Within each section, all islets containing approximately ≥ 20 endocrine cells were co-imaged for syntaxin-4, glucagon, and the presence of insulin. The absence of insulin in the islets of diabetic donors was also recorded. Image sets (syntaxin-4, ±insulin, and glucagon) were merged using Adobe Photoshop CS6 (Adobe Systems, San Jose, CA, USA). In the merged figure illustrations described under “Results”, glucagon-immunostained cells (initially brown) were converted to a cyan pseudo-colour prior to superimposition with fluorescently labelled syntaxin-4 and insulin.

## Image analysis

Islets from each donor section were enumerated and classified as insulin-positive or negative, and syntaxin-4 expression was subsequently evaluated within both islet types. In some islets from diabetic cases, where insulin cells were scarce, more than 2 insulin-positive cells per islet were analysed for syntaxin-4 intensities. Identification and enumeration of such cells were achieved by the lack of staining in their corresponding nuclei. The overall IF intensities for syntaxin-4 in cells from insulin-positive or -negative areas within each islet were quantified using ImageJ software, as previously described (Nakayasu et al. 2020; Sun et al. 2016).

Briefly, a freehand selection tool was used to outline syntaxin-4-stained cells within islet areas corresponding to insulin positivity in 60 randomly selected islets from each non-diabetic donor (Group 1 and 2). Background intensities were determined from the exocrine area immediately adjacent to each islet. For the diabetic cohorts, where insulin-positive cells were often reduced or dispersed, multiple cellular aggregates were outlined within each islet using the same freehand tool. Notably, fewer islets were analyzed in the diabetic groups due to the variable and limited presence of insulin-positive islets.

The net IF intensity of syntaxin-4 within each islet was calculated using the Corrected Total Cell Fluorescence (CTCF) formula:

$$ \begin{aligned} {\mathrm{CTCF}} = & {\mathrm{Integrated}}\;{\mathrm{Density}} - \left( {{\mathrm{Area}}\;{\mathrm{of}}\;{\mathrm{selected}}\;{\mathrm{background}}\;{\mathrm{region}}} \right. \\ & \left. { \;\, \times {\mathrm{mean}}\;{\mathrm{fluorescence}}\;{\mathrm{intensity}}\;{\mathrm{of}}\;{\mathrm{background}}} \right) \\ \end{aligned} $$

Due to the presence of insulin-negative islets in the two diabetic groups, syntaxin-4 intensities were derived from a variable number of remaining insulin-positive islets, as detailed in ESM Table 1.

### Statistical analysis

Statistical analyses were performed using Python (version 3.12.3). Normality of data distribution was assessed using the Shapiro-Wilk test. For non-normally distributed data, comparisons between more than two groups were conducted using the Kruskal-Wallis test followed by Dunn’s post hoc test, and comparison between two groups was performed using the Mann-Whitney U test. For normally distributed data, comparison between two groups was conducted using the Student’s *t*-test. *P* ≤ 0.05 was considered statistically significant.

## Results

### Percentage of insulin-positive islets

The overall percentages of insulin-positive islets in the 4 study groups are summarised in Table 4 (values per case are itemised in ESM Table 1). In the non-diabetic autoantibody-negative donors, all islets were insulin-positive, while in the non-diabetic autoantibody-positive donors, all donor islets were insulin-positive, except in nPOD 6267, where a single islet was insulin-negative. In newly-diagnosed diabetic group, the percentage of insulin-positive islets per donor ranged from 7.41 to 69.33% (ESM Table 1c), with a combined percentage of 30.6%. In the long-term diabetic group, insulin-negative islets were present in 3/10 donors, while in the remaining 6 donors, the percentage of insulin-positive islets ranged from 1.7% (nPOD 6088 T1D 5 years) to 96.55% (nPOD 6551; T1D 0.58 year; ESM Table 1d). The combined percentage of insulin-positive islets in this group was 21% (Table 4).

**Table 4:** Cumulative percentage of insulin-positive islets in all donors per study group

Study group	Number of donors	Total number of islets examined	Percentage (%) of insulin-positive islets
Group 1: Non-diabetic AAb-negative (nPOD)	7	875	100
Group 2: Non-diabetic AAb positive (nPOD)	6	461	99.8
Group 3: New-onset T1D (DiViD)	4	278	30.6
Group 4: Long term T1D (nPOD)	10	747	21.0

AAb, autoantibody; DiViD, Diabetes Virus Detection; nPOD, Network for Organ Donors with Diabetes. Please refer to ESM Table 1 for detailed information for each donor

## IHC specificity of rabbit anti-syntaxin-4

In the IF procedure for syntaxin-4, substitution of anti-rabbit syntaxin-4 with antibody diluent or normal rabbit IgG showed an absence of staining of islet cells and the surrounding region when compared with adjacent sections exposed to anti-syntaxin-4 (ESM Fig. 1). The same IF procedure for syntaxin-4 when applied to non-diabetic mouse, rat and human pancreatic sections, resulted in staining of beta cells but not glucagon cells (ESM Fig. 2a-l). As a further test for the IHC specificity of the anti-syntaxin-4 antibody, immunostaining of human tonsil sections using the same procedure resulted in negative staining for syntaxin-4 but positive staining for human CD45 cells following dual staining with anti-CD45 (ESM Fig. 3a, b).

**Fig. 1:**
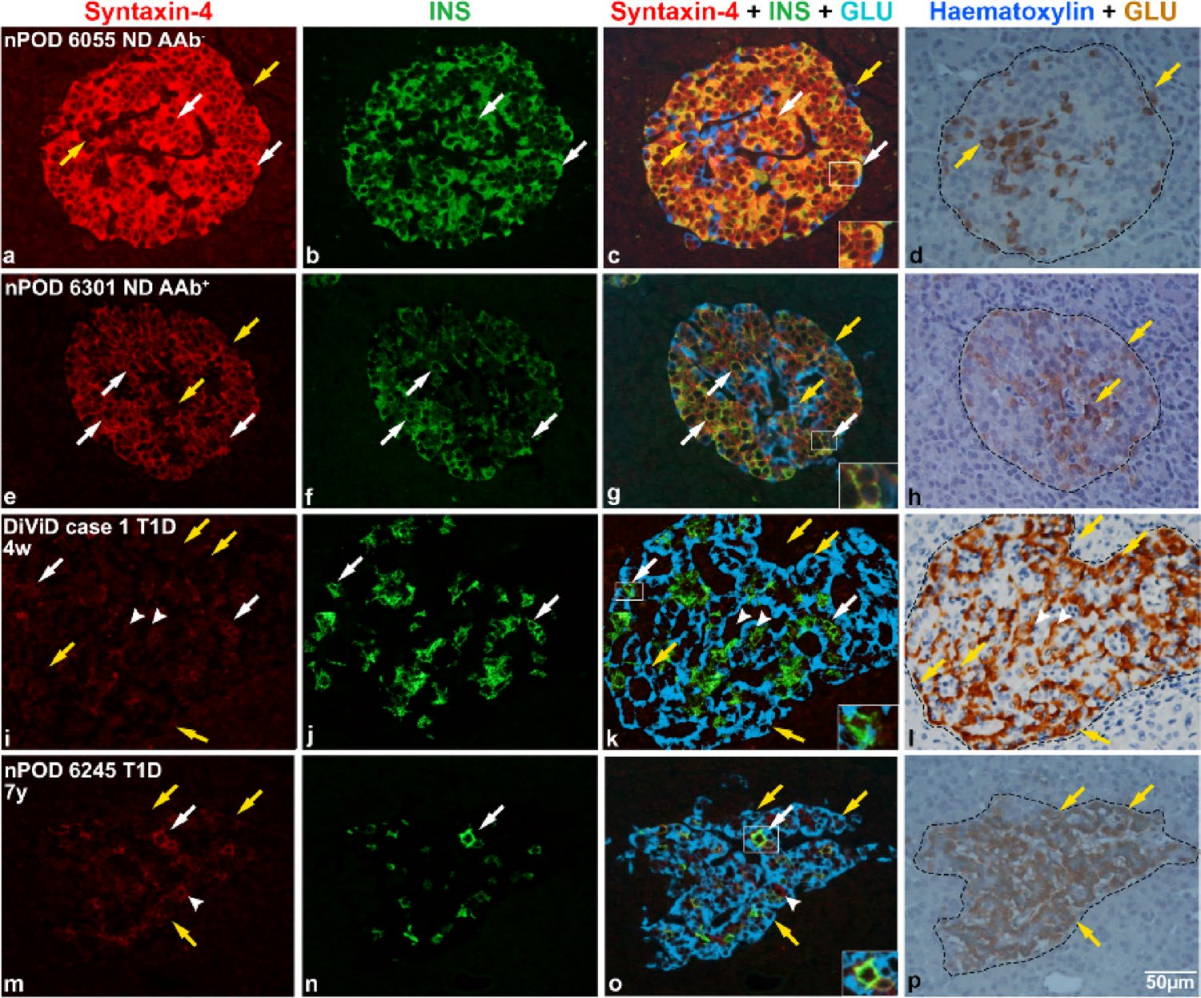
Immunohistochemical analysis of pancreas sections showing islets with syntaxin-4, insulin and glucagon staining in the 4 study groups. Column 1: syntaxin-4; Column 2: insulin; Column 3: merged view of syntaxin-4 and insulin by dual immunofluorescence and glucagon by immunoperoxidase (Brown positive cells converted to cyan); Column 4: haematoxylin counterstaining with islet boundaries marked by black dashed lines. (**a-d**) Group 1: non-diabetic autoantibody-negative donor. (**e-h**) Group 2: non-diabetic autoantibody-positive donor. (**i-l**) Group 3: newly diagnosed donor. (**m-p**) Group 4: longer term diabetic donor. White arrows indicate syntaxin-4 in insulin-positive cells; yellow arrows indicate glucagon cells; white arrowheads indicate syntaxin-4 in cells negative for insulin and glucagon. The insets in Column 3 show magnified views of cells from each donor that are positive for syntaxin-4 and insulin. Scale bar = 50 μm (applies to all panels). AAb^+^, autoantibody positive; DiViD, Diabetes Virus Detection; GLU, glucagon; INS, insulin; ND, non-diabetic; nPOD, Network for Pancreatic Organ Donors with Diabetes; T1D, type 1 diabetes

**Fig. 2:**
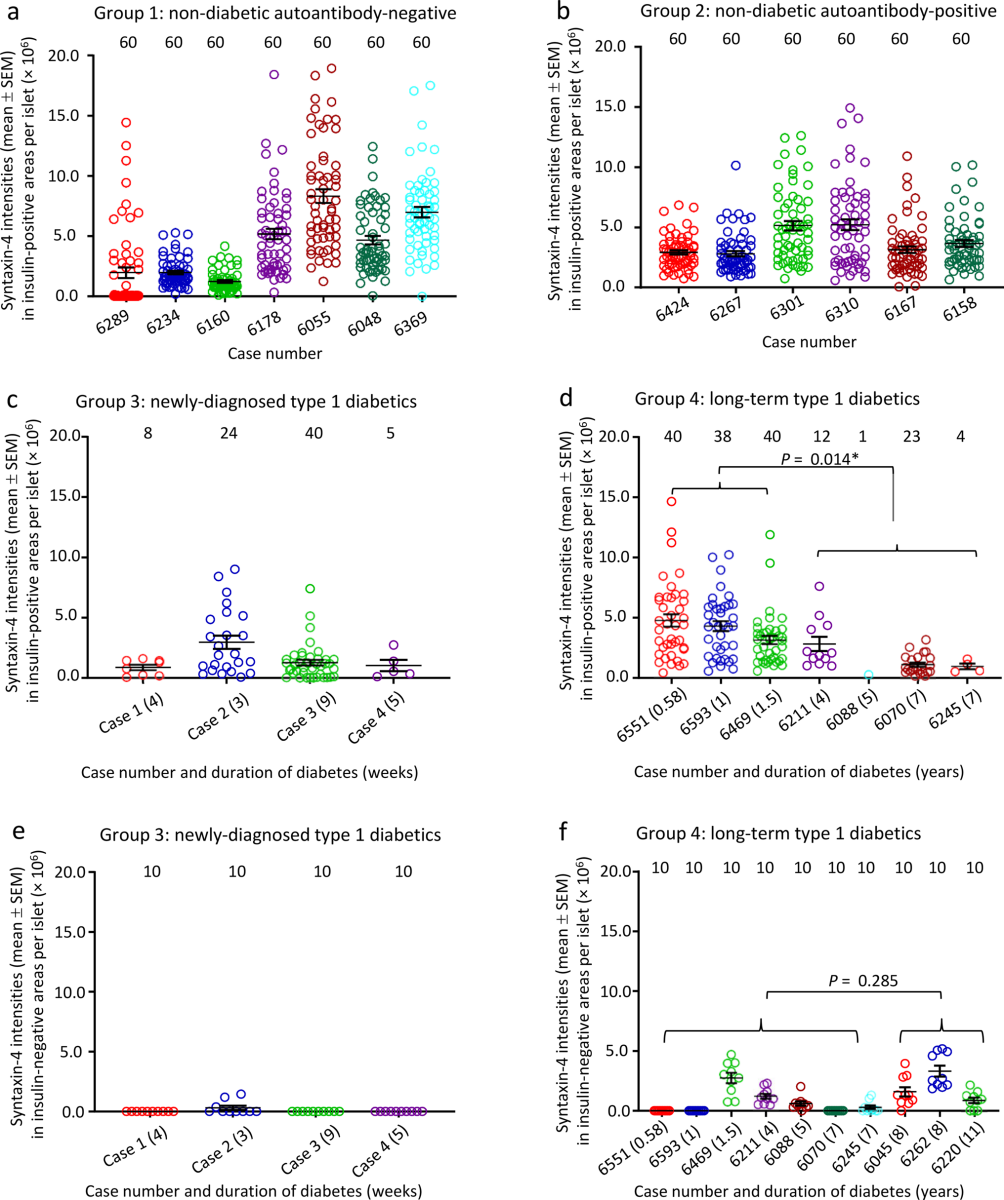
Syntaxin-4 intensities in randomly selected islets across the study groups. (**a-f**) Syntaxin-4 intensities were expressed in arbitrary units (Mean ± SEM) per donor in insulin-positive areas from the 4 groups (**a-d**), and in insulin-negative areas from the 2 diabetic groups (**e**, **f**). Islets from donors 6045, 6262 and 6220 were insulin-negative in Group 4 and were excluded in (**d**). The number of islets analysed is indicated at the top of the graph for each donor. Comparisons were conducted using Student’s t-test. *P* values are indicated above the bars, and the significance difference is denoted by “*” (*p* < 0.05)

**Fig. 3:**
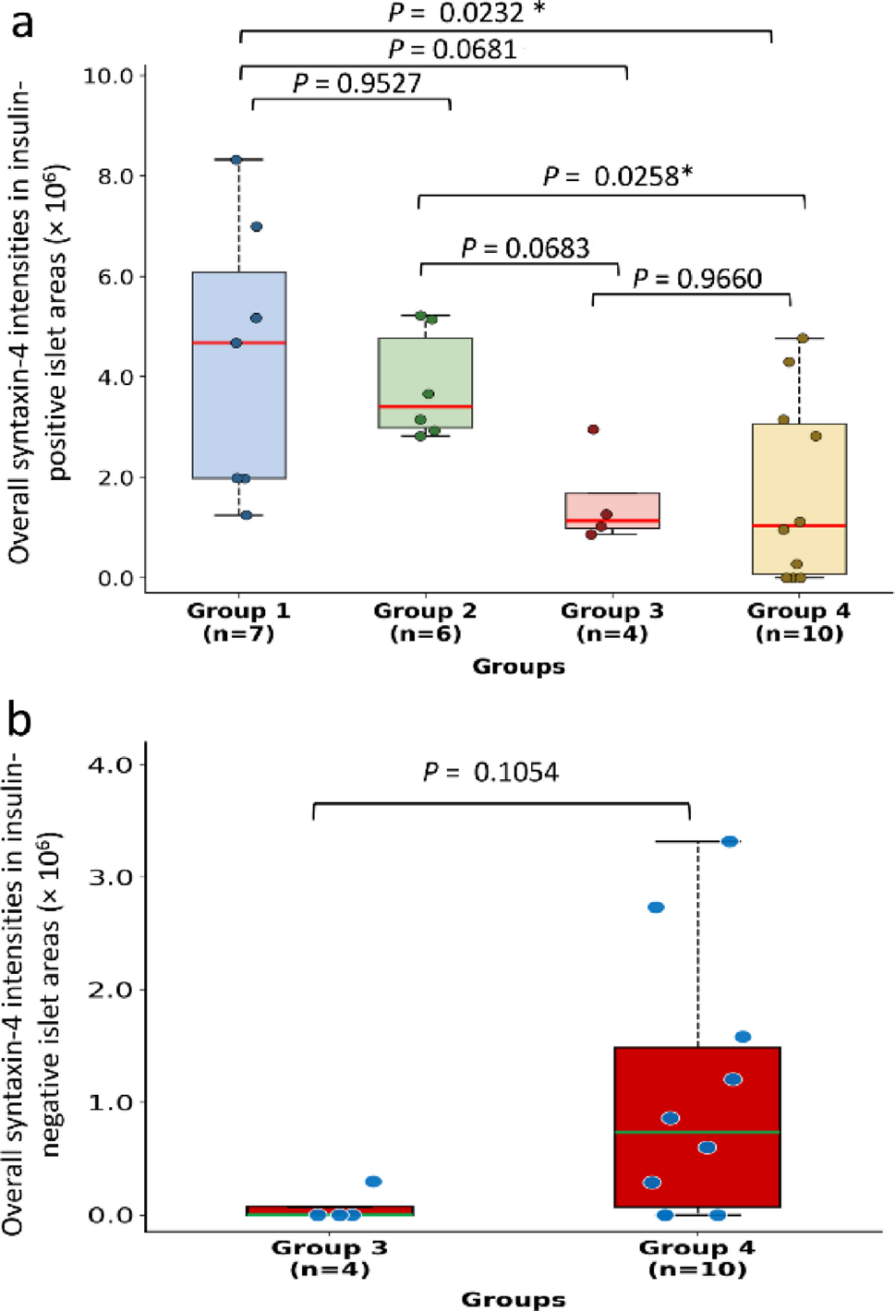
Overall syntaxin-4 intensities in insulin-positive areas of islets from the 4 groups (**a**), and insulin-negative islet areas from the 2 diabetic groups (**b**). Dots represent the mean value of syntaxin-4 intensities per donor as shown in Fig. 2. Box plots show the median (horizontal line), interquartile range (box), and 1.5 × Interquartile range (IQR) (whiskers). Comparisons were conducted using the Kruskal-Wallis test followed by Dunn’s post hoc test (**a**) and Mann-Whitney U test (**b**), respectively. *P* values are indicated above the bars, and the significance difference is denoted by “*” (*p* < 0.05)

### Distribution and localisation of syntaxin-4 in islet cells

Examples of the overall cellular distribution of syntaxin-4 and its co-localisation from a representative islet from each study group are shown in Fig. 1. Images of additional islets from the 4 groups stained similarly are shown in ESM Fig. 4–8. In all the assembled photomicrographs, the first column shows syntaxin-4 in islet cells. The second column depicts corresponding islets co-stained for insulin, and the third column shows merged images of syntaxin-4, insulin and glucagon. The fourth column illustrates corresponding islets and the surrounding acinar area following counterstaining with haematoxylin.

Among non-diabetic nPOD donors with and without islet cell autoantibodies, the presence of syntaxin-4 was observed in all insulin-positive cells, with some variability in cell intensities (Fig. 1a-h; ESM Fig. 4a-l; ESM Fig. 5a-l). Thus, most insulin-positive islets showed moderate to strong staining intensities for syntaxin-4, with weaker staining in some islets (nPOD 6160, ESM Fig. 4i-l).

In DiViD case 1, a minority of islets positive for insulin showed an absence or weak staining for syntaxin-4 (Fig. 1i-l; ESM Fig. 6e-h). In contrast, strong staining for syntaxin-4 was observed in insulin-positive cells from DiViD case 2 (ESM Fig. 6a-d). However, some islets from the same case expressing a considerably reduced number of insulin cells also showed syntaxin-4 expression in a few insulin- and glucagon-negative cells (ESM Fig. 6e-h). In DiViD case 3, weaker staining for syntaxin-4 was observed in insulin-positive cells (ESM Fig. 6i-l). In DiViD case 4, weak staining for syntaxin-4 was also observed in a minority of islets that showed a reduced number of insulin-positive cells (results not shown).

Representative islets from donors with longer duration of T1D co-stained for syntaxin-4, insulin and glucagon and their merged images are shown in Fig. 1m-p and ESM Fig. 7a-x and ESM Fig. 8a-p). In nPOD 6551 (0.58 years of T1D) and nPOD 6593 (1.58 years of T1D), strong staining for syntaxin-4 was observed in insulin cells, although a few cells negative for insulin were positive for syntaxin-4 (ESM Fig. 7a-h). The presence of syntaxin-4 in cells negative for insulin was also observed in nPOD 6469 (ESM Fig. 7m-p; 1.5 years of T1D) but not in nPOD 6211 (ESM Fig. 7q-t; 4 years of T1D). In donor 6088 (5 years of T1D), several cells negative for insulin and glucagon showed positive staining for syntaxin-4 (ESM Fig. 7u-x). This staining pattern was also observed in donors 6245 (Figs. 1m-p, 7 years of T1D), 6262 (ESM Fig. 8i- l, 8 years of T1D), and 6220 (ESM Fig. 8m-p, 11 years of T1D). In nPOD 6070 (7 years of T1D), syntaxin-4 was present in mostly insulin-positive cells (ESM Fig. 8a-h).

### Determination of staining intensities for syntaxin-4

Quantified syntaxin-4 intensities in insulin-positive regions of randomly selected islets are presented in Fig. 2a-d. Concurrently, syntaxin-4 expression profiles in insulin-negative islet areas from diabetic donors (Groups 3 and 4) are depicted in Fig. 2e, f. The intensity values are also shown in tabular form (ESM Tables 2a-f). Donors from the 4 study groups showed marked variation in individual islet intensities (within insulin-positive areas) both among islets from the same donor and between donors. In the long-term diabetic group, syntaxin-4 intensity in insulin-positive areas was significantly lower in donors with 4–7 years of T1D than in those with shorter T1D duration (Fig. 2d). These variations were less marked in insulin-negative areas of islets in groups 3 and 4 (Fig. 2e, f). An increase in syntaxin-4 intensity in insulin-negative areas was observed in donors with 8 and 11 years of T1D compared with those with shorter duration, but this trend did not reach statistical significance (Fig. 2f).

Mean syntaxin-4 intensities per donor are listed in ESM Table 2, and the mean intensities per study group are indicated in Table 3. Box plots compare intensities in insulin-positive areas across all four groups (Fig. 3a) and in insulin-negative areas for the two diabetic groups (Fig. 3b). In insulin-positive areas, syntaxin-4 intensities differed significantly between groups 1 vs. 4 (*p* = 0.023), 2 vs. 4 (*p* = 0.026), and showed a trend for groups 1 vs. 3 and 2 vs. 3 (both *p* = 0.068), but not between groups 3 and 4 (*p* = 0.966). No significant difference was found in insulin-negative areas between groups 3 and 4 (*p* = 0.966).

## Discussion

This study has analysed syntaxin-4 levels in islets from human donors in 4 distinct study groups with and without T1D. Syntaxin-4 was co-localised with insulin-expressing endocrine cells and in some islet cellsthat were negative for insulin and glucagon. The overall mean intensities of syntaxin-4 in insulin-positive areas of islets from non-diabetic autoantibody-negative and -positive donor groups were similar but higher than those in newly diagnosed and longer-term diabetic donors. However, the intensities within individual islets across donors showed marked intra- and inter-islet variation, irrespective of diabetes status. This observation is consistent with previous studies, which demonstrate considerable beta cell heterogeneity within an islet and among islets from non-diabetic subjects (Benninger and Piston 2014; Miranda et al. 2021). It is also in agreement with our recently reported observation of inter- and intra-islet heterogeneity in the expression of glutathione peroxidase-1 in diabetic and non-diabetic human donors (Pala et al. 2025). Islet-to-islet heterogeneity of syntaxin-4 levels within a donor may be attributed to several factors, such as non-uniform capillary islet innervation, islet size, pancreatic location, and metabolic differences among individual beta cells (Dybala and Hara, 2019; Wang et al, 2014). Intra-islet functional heterogeneity, such as asynchronous secretion of insulin in response to glucose, beta cell dedifferentiation, transdifferentiation, and senescence among individual beta cells, may also be contributing factors (Álvarez-Cubela et al. 2025; Cinti et al. 2016; Thompson et al. 2023; Wang et al. 2014).

A similar level of syntaxin-4 intensity in autoantibody-negative and positive non-diabetic donors shown in this study, suggests that, in both groups, the final exocytosis step is maintained. However, analysis of pancreatic sections from a larger number of non-diabetic donors with multiple autoantibodies of differing titres, coupled with known polygenic risk markers and evidence of beta-cell dysfunction, is warranted to provide a more precise assessment of syntaxin-4 levels in this important group. Our results showing lower syntaxin-4 intensities in three of four newly diagnosed donors compared with nPOD donors at 0.58, 1, and 1.5 years are notable and may reflect glycaemic, metabolic, and physiological differences between donors in the DiViD Study and nPOD. In addition, our results demonstrate that syntaxin-4 intensity in insulin-positive cells is lower in donors with longer disease duration (4–7 years) than in nPOD donors with shorter disease duration. These results suggest that the final exocytotic step is likely to be impaired in insulin-positive islets from some subjects with a longer duration of T1D.

The current study highlights differences from a previously reported brief study that showed lower cumulative mean syntaxin-4 intensities in insulin-positive islets from T1D donors than in those from non-diabetic donors (Oh et al. 2014). However, in the previous study, the authors analysed a much smaller number of islets from only 3 younger subjects with T1D and compared their intensities with those of 4 non-diabetic subjects, which may constitute a different endotype from the current cohorts. In the present study, analysis was performed on a much larger number of islets and from older subjects (19–44 years) and included rare diabetic donors with new-onset and longer duration of disease. Such differences in donor characteristics may have contributed to the variable results obtained in the two studies.

Our unexpected finding that syntaxin-4 is also expressed in insulin- and glucagon-negative cells in islets from some diabetic subjects, particularly in donors with longer disease duration, is of interest and has not been reported previously. We suggest that syntaxin-4 may be expressed in dedifferentiated beta cells lacking insulin, or in other endocrine cell types such as somatostatin, ghrelin, or pancreatic polypeptide cells within the islet. However, previous studies have shown that in some subjects with T1D, selective islet cells may harbour extremely low levels of insulin that are below the detection limit of current IHC approaches but can be detected by an IPO approach involving microscopic image acquisition with prolonged exposure to white light (Lam et al. 2019). Our analysis showing a qualitative trend towards higher syntaxin-4 intensities in insulin- and glucagon-negative cells in donors with 8–11 years of T1D warrants future analysis from a larger cohort of donors from this group.

We are cognizant of some limitations of the present study, including its unavoidable cross-sectional design. The impact of co-existing insulitis on syntaxin-4 expression in islets of diabetic subjects is unknown. Although IHC approaches may have limitations, such as limited sensitivity, they circumvent tissue disruption, enabling reliable identification of spatially distributed cellular proteins utilizing co-localisation techniques. Studies employing more sensitive approaches, such as imaging mass spectrometry, spatial proteomics, and time-lapse studies of human islets stimulated with glucose in culture, may contribute to a more precise understanding of the role of syntaxin-4 and its partner proteins in facilitating insulin release from beta-cell granules.

Extrapolation of our current findings to all subjects with recent and long-term T1D is not appropriate due to differences in age of onset, unique disease trajectories, and known variabilities in the molecular and cellular immunopathology of islets in affected individuals. We are aware that while DiViD donors were better optimised for diabetes control during and prior to the biopsy procedure, some deceased donors from nPOD may have experienced unavoidable delays during and prior to pancreas retrieval.

Despite the above limitations, the present studies on rare human pancreatic tissues provide new insights into the comparative expression of syntaxin-4 in diabetic and non-diabetic donors, with and without islet cell antibodies, and highlight islet beta-cell heterogeneity in syntaxin-4 levels. Further studies utilising pancreas from larger donor cohorts are likely to contribute to a more detailed understanding of the critical role of syntaxin-4 in promoting insulin release via exocytosis.

## Supplementary Information

Below is the link to the electronic supplementary material.

Supplementary Material 1

## Data Availability

All data supporting the findings of this study are available within the paper and its Supplementary Information. Additional data are available from the corresponding author on request.
