# Supplementary material for: Syntaxin-4, a key exocytosis mediating protein, shows heterogeneous expression in insulin-positive cells of human donors with new-onset and longer duration of type 1 diabetes: comparison with non-diabetic autoantibody-positive and -negative donors
Source: J Mol Histol. 2026 Jul 30;57(4):252. doi: 10.1007/s10735-026-10901-4 (PMC13423997; doi:10.1007/s10735-026-10901-4)
Supplement: Supplementary file 1 — Supplementary Material 1 [file 10735_2026_10901_MOESM1_ESM.pdf]

Supplementary Materials

ESM Table 1a. Group 1 (non-diabetic autoantibody-negative): Enumeration of insulin-positive islets per donor

Case ID (nPOD), disease and autoantibody (AAb) status	Number of islets examined per donor	Number of insulin-positive islets per donor	% of insulin-positive islets per donor
nPOD 6289, non-diabetic, AAb-negative	128	128	100
nPOD 6234, non-diabetic, AAb-negative	109	109	100
non-diabetic, AAb-negative	175	175	100
nPOD 6178, non-diabetic, AAb-negative	167	167	100
nPOD 6055, non-diabetic, AAb-negative	133	133	100
nPOD 6048, non-diabetic, AAb-negative	77	77	100
nPOD 6369, non-diabetic, AAb-negative	86	86	100

ESM Table 1b. Group 2 (non-diabetic autoantibody-positive): Enumeration of insulin-positive islets per donor

Case ID (nPOD), disease and autoantibody (AAb) status	Number of islets examined per donor	Number of insulin-positive islets per donor	% of insulin-positive islets per donor
nPOD 6424, non-diabetic, 2AAb	74	74	100
nPOD 6267, non-diabetic, 2AAb	67	65	97
nPOD 6301, non-diabetic, 1AAb	56	56	100
nPOD 6310, non-diabetic, 1AAb	84	84	100
nPOD 6167, non-diabetic, 2AAb	85	85	100
nPOD 6158, non-diabetic, 2AAb	95	95	100

ESM Table 1c. Group 3 (newly-diagnosed T1D): Enumeration of insulin-positive islets per donor

Case ID (nPOD), disease and autoantibody (AAb) status	Number of islets examined per donor	Number of insulin-positive islets per donor	% of insulin-positive islets per donor
DiViD Case 1, 4 weeks, 4 AAb	47	8	17.02
DiViD Case 2, 3 weeks, 3 AAb	48	17	35.41
DiViD Case 3, 9 weeks, 3 AAb	75	52	69.33
DiViD Case 4, 5 weeks, 3 AAb	108	8	7.41

ESM Table 1d. Group 4 (long-term T1D): Enumeration of insulin-positive islets per donor

**Case ID (nPOD), disease and autoantibody (AAb) status**	**Number of islets examined per donor**	**Number of insulin-positive islets per donor**	**% of insulin-positive islets per donor**
nPOD 6551, 0.58 year, 4 AAb	58	56	96.55
nPOD 6593, 1 year, 4 AAb	39	34	87.18
nPOD 6469, 1.5 years, 1 AAb	125	20	16.00
nPOD 6211, 4 years, 4 AAb	64	12	17.19
nPOD 6088, 5 years, 4 AAb	59	1	1.70
nPOD 6070, 7 years, 2 AAb	92	28	30.43
nPOD 6245, 7 years,2 AAb	106	6	5.66
nPOD 6045, 8 years, 2 AAb	66	0	0
nPOD 6262, 8 years, 3 AAb	98	0	0
nPOD 6220, 11 years, 2 AAb	40	0	0

ESM Table 2a. Group 1: Non-diabetic autoantibody-negative donors from nPOD, showing mean ± SEM syntaxin-4 staining intensities (arbitrary units) in the insulin-positive area of the islets per donor

Case ID (nPOD), disease and autoantibody status	Number of insulin-positive islets analysed for syntaxin-4	Mean syntaxin-4 staining intensities ± SEM in insulin-positive areas of islets per donor
nPOD 6289, non-diabetic, AAb-negative	60	$1.971 \times 10^6 \pm 4.426 \times 10^5$
nPOD 6234, non-diabetic, AAb-negative	60	$1.979 \times 10^6 \pm 1.494 \times 10^5$
nPOD 6160, non-diabetic, AAb-negative	60	$1.245 \times 10^6 \pm 1.106 \times 10^5$
nPOD 6178, non-diabetic, AAb-negative	60	$5.175 \times 10^6 \pm 4.382 \times 10^5$
nPOD 6055, non-diabetic, AAb-negative	60	$8.323 \times 10^6 \pm 5.677 \times 10^5$
nPOD 6048, non-diabetic, AAb-negative	60	$4.675 \times 10^6 \pm 3.496 \times 10^5$
nPOD 6369, non-diabetic, AAb-negative	60	$6.991 \times 10^6 \pm 4.317 \times 10^5$

ESM Table 2b. Group 2: Non-diabetic autoantibody-positive doors from nPOD, showing mean ± SEM syntaxin-4 staining intensities (arbitrary units) in insulin-positive areas of islets per donor

Case ID (nPOD), disease and autoantibody status	Number of insulin-positive islets analysed for syntaxin-4	Mean syntaxin-4 staining intensities ± SEM in insulin-positive areas of islets per donor
nPOD 6424, non-diabetic, 2AAb	60	$2.927 \times 10^6 \pm 1.782 \times 10^5$
nPOD 6267, non-diabetic, 2AAb	60	$2.817 \times 10^6 \pm 2.186 \times 10^5$
nPOD 6301, non-diabetic, 1AAb	60	$5.142 \times 10^6 \pm 3.907 \times 10^5$
nPOD 6310, non-diabetic, 1AAb	60	$5.219 \times 10^6 \pm 4.438 \times 10^5$
nPOD 6167, non-diabetic, 2AAb	60	$3.145 \times 10^6 \pm 2.783 \times 10^5$
nPOD 6158, non-diabetic, 2AAb	60	$3.655 \times 10^6 \pm 2.725 \times 10^5$

ESM Table 2c. Group 3: Newly-diagnosed cases from DiViD, showing syntaxin-4 staining intensities (arbitrary units) in insulin-positive area of islets

Case ID (DiViD), disease duration and AAb status	Number of insulin-positive islets analysed for syntaxin-4	Mean syntaxin-4 staining intensities ± SEM in insulin-positive area of islets per donor
DiViD Case 1, 4 weeks, 4 AAb	8	$8.396 \times 10^5 \pm 2.291 \times 10^5$
DiViD Case 2, 3 weeks, 3 AAb	24	$2.950 \times 10^6 \pm 5.541 \times 10^5$
DiViD Case 3, 9 weeks, 3 AAb	40	$1.405 \times 10^6 \pm 2.349 \times 10^5$
DiViD Case 4, 5 weeks, 3 AAb	5	$1.015 \times 10^6 \pm 4.780 \times 10^5$

ESM Table 2d. Group 4: Long-term diabetic cases from nPOD, showing syntaxin-4 staining intensities (arbitrary units) in insulin-positive area of islets

Case ID 9nPOD), disease duration and AAb status	Number of insulin-positive islets analysed for syntaxin-4	Mean syntaxin-4 staining intensities ± SEM in insulin-positive area of islets per donor
nPOD 6551, 0.58 year, 4 AAb	40	$4.763 \times 10^6 \pm 5.083 \times 10^5$
nPOD 6593, 1 year, 4 AAb	38	$4.293 \times 10^6 \pm 4.132 \times 10^5$
nPOD 6469, 1.5 years, 1 AAb	40	$3.146 \times 10^6 \pm 3.467 \times 10^5$
nPOD 6211, 4 years, 4 AAb	12	$2.815 \times 10^6 \pm 5.927 \times 10^5$
nPOD 6088, 5 years, 4 AAb	1	2.696×10^5
nPOD 6070, 7 years, 2 AAb	23	$1.107 \times 10^6 \pm 1.703 \times 10^5$
nPOD 6245, 7 years, AAb	4	$1.005 \times 10^6 \pm 2.456 \times 10^5$
nPOD 6045, 8 years, 2 AAb	0	0
nPOD 6262, 8 years, 3 AAb	0	0
nPOD 6220, 11 years, 2 AAb	0	0

ESM Table 2e. Group 3: Newly-diagnosed cases from DiViD, showing syntaxin-4 staining intensities (arbitrary units) in insulin-negative area of islets

Case ID (DiViD), disease duration and AAb status	Number of insulin-negative islets analysed for syntaxin-4	Mean syntaxin-4 staining intensities ± SEM in insulin-negative area of islets per donor
DiViD Case 1, 4 weeks, 4 AAb	10	$3.008x10^5 \pm 1.742x10^5$
DiViD Case 2, 3 weeks, 3 AAb	10	0
DiViD Case 3, 9 weeks, 3 AAb	10	0
DiViD Case 4, 5 weeks, 3 AAb	10	0

ESM Table 2f. Group 4: Long-term diabetic cases from nPOD, showing syntaxin-4 staining intensities (arbitrary units) in insulin-negative area of islets

Case ID (nPOD), disease duration and AAb status	Number of insulin-negative islets analysed for syntaxin-4	Mean syntaxin-4 staining intensities ± SEM in insulin-negative area of islets per donor
nPOD 6551, 0.58 year, 4AAb	10	0
nPOD 6593, 1 year, 4AAb	10	0
nPOD 6469, 1.5 years, 1AAb	10	$2.737x10^6 \pm 4.277x10^5$
nPOD 6211, 4 years, 4AAb	10	$1.206x10^6 \pm 2.063x10^5$
nPOD 6088, 5 years, 4AAb	10	$6.014x10^5 \pm 1.714x10^5$
nPOD 6070, 7 years, 2AAb	10	0
nPOD 6245, 7 years, 2AAb	10	$2.912x10^5 \pm 1.494x10^5$
nPOD 6045, 8 years, 2AAb	10	$1.583x10^6 \pm 3.916x10^5$
nPOD 6262, 8 years, 3AAb	10	$3.318x10^6 \pm 4.510x10^5$
nPOD 6220, 11 years, 2AAb	10	$8.642x10^5 \pm 2.273x10^5$

ESM Table 3a. Overall mean syntaxin-4 intensities (arbitrary units) in insulin-positive area of islets per study group

Group	Mean syntaxin-4 staining intensities ± SEM in insulin-positive areas of islets per study group
1	$4.337 \times 10^6 \pm 1.907 \times 10^5$
2	$3.791x10^6 \pm 2.373x10^5$
3	$1.773x10^6 \pm 3.384x10^5$
4	$1.936x10^6 \pm 3.224x10^5$

ESM Table 3b. Overall mean syntaxin-4 intensities (arbitrary units) in insulin-negative area of islets in groups 3 and 4 (diabetic groups)

Group and disease status	Mean syntaxin-4 staining intensities ± SEM in insulin-negative area of islets per study group
3 (newly-diagnosed diabetes)	$3.008x10^5 \pm 1.742x10^5$
4 (long-term diabetes)	$1.514x10^6 \pm 1.688x10^5$

ESM Figure 1

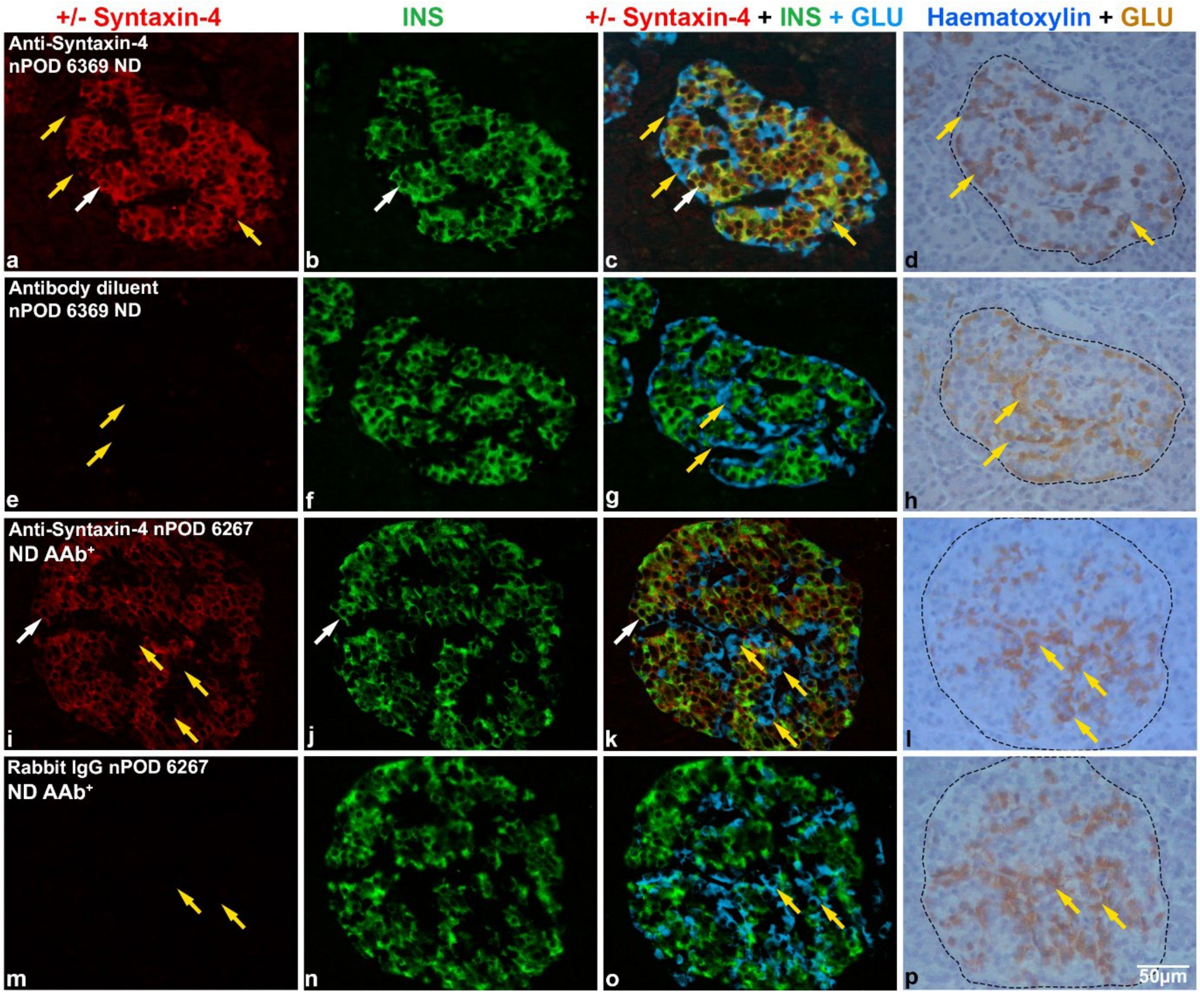

ESM Fig. 1 Immunohistochemical specificity of rabbit anti-syntaxin-4 tested on human pancreatic sections from non-diabetic nPOD donors by replacing anti-syntaxin-4 with antibody diluent or normal rabbit IgG in the immunohistochemical procedure. (a) Addition of anti-syntaxin-4 to a pancreatic section from nPOD 6369; (b) Same section as (a) co-stained for insulin. (c) Merged view of syntaxin-4 + insulin + glucagon (stained by immunoperoxidase and glucagon-positive cells converted to a cyan colour). (d) corresponding section counterstained by haematoxylin where the islet boundary in indicated as black dashes. (e) Addition of antibody diluent to adjacent section in the procedure for syntaxin-4 staining. (f) Same section as (e) co-stained for insulin. (g) Merged view of insulin and glucagon (stained by immunoperoxidase and glucagon-positive cells converted to a cyan colour). (h) Corresponding section following counterstaining with haematoxylin where the islet boundary is denoted by black dashes. (i) Addition of anti-syntaxin-4 to a pancreatic section from nPOD 6267. (j) Same section as in (i) co-stained for insulin. (k) Merged view of syntaxin-4 + insulin + glucagon (stained by immunoperoxidase and glucagon-positive cells converted to a cyan colour). (l) corresponding islet counterstained with haematoxylin where the islet boundary is denoted by black dashes. (m) Addition of normal rabbit IgG to an adjacent section from nPOD 6267 in the procedure for syntaxin-4 staining. (n) Same section as (m) co-stained for insulin. (o) Merged view of insulin + glucagon (stained by immunoperoxidase and glucagon-positive cells converted to a cyan colour). (p) Corresponding section counterstained with haematoxylin where the islet boundary is denoted by black dashes. White arrows indicate syntaxin-4 in insulin-positive cells; yellow arrows indicate glucagon cells. Scale bar in (p) 50 μm, applies to all micrographs. AAb^{+}, autoantibody-positive; GLU, glucagon; INS, insulin; ND, non-diabetic; nPOD, Network for Pancreatic Organ Donors with Diabetes

ESM Figure 2

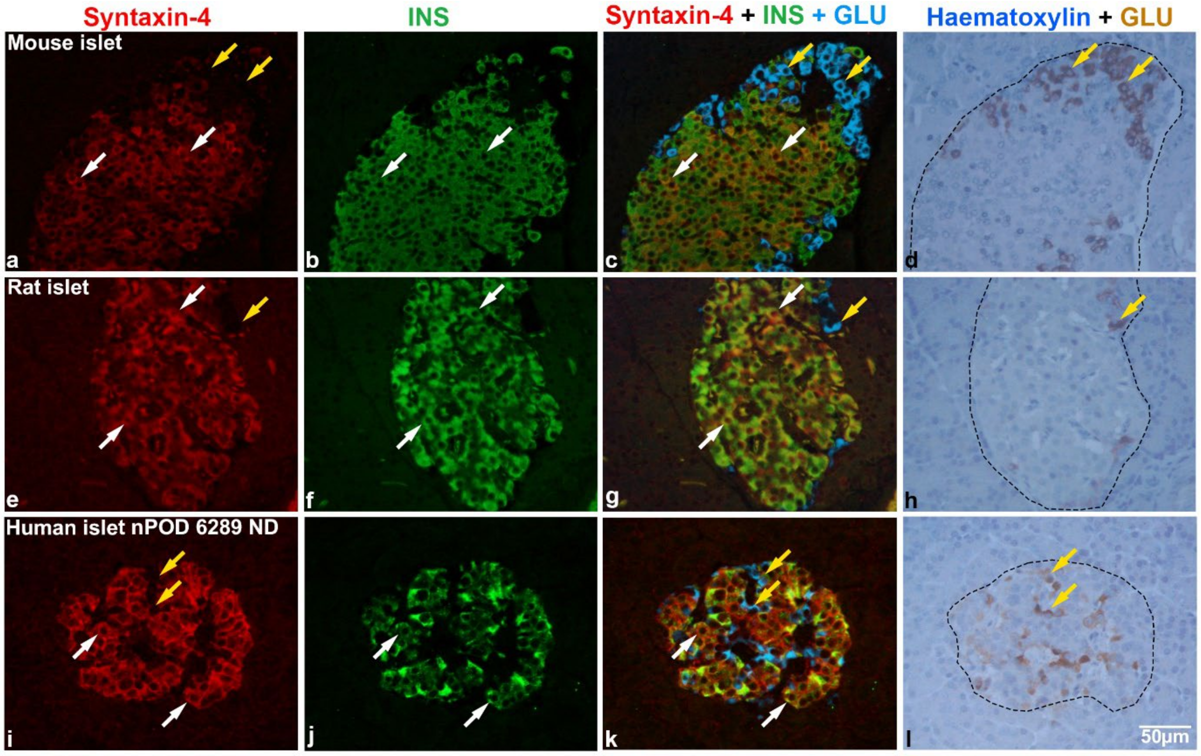

ESM Fig. 2 Immunohistochemical cross-reactivity of anti-syntaxin-4 in islet cells of mouse, rat and human pancreatic sections. First column: syntaxin-4; second column: insulin; third column shows a merged view of syntaxin-4, insulin and glucagon (following conversion of brown glucagon-positive cells to a cyan colour). The fourth column shows corresponding sections counterstained with haematoxylin where the islet boundaries are indicated by black dashes. White arrows indicate syntaxin-4 in insulin-positive cells; yellow arrows indicate glucagon cells. Scale bar in (l) 50 µm, applies to all micrographs. GLU, glucagon; INS, insulin; ND, non-diabetic; nPOD, Network for Pancreatic Organ Donors with Diabetes.

ESM Figure 3

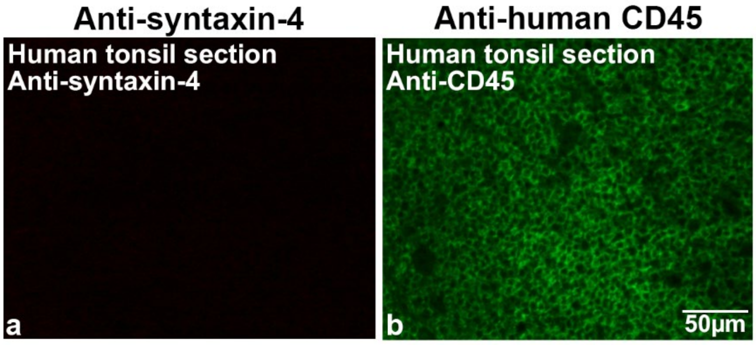

ESM Fig. 3 Application of syntaxin-4 immunofluorescence protocol to human tonsil section. (a) Negative staining for syntaxin-4. (b) Corresponding field shows positive staining for human CD45 cells (green). Scale bar in (b) 50 µm, also applies to (a).

ESM Figure 4

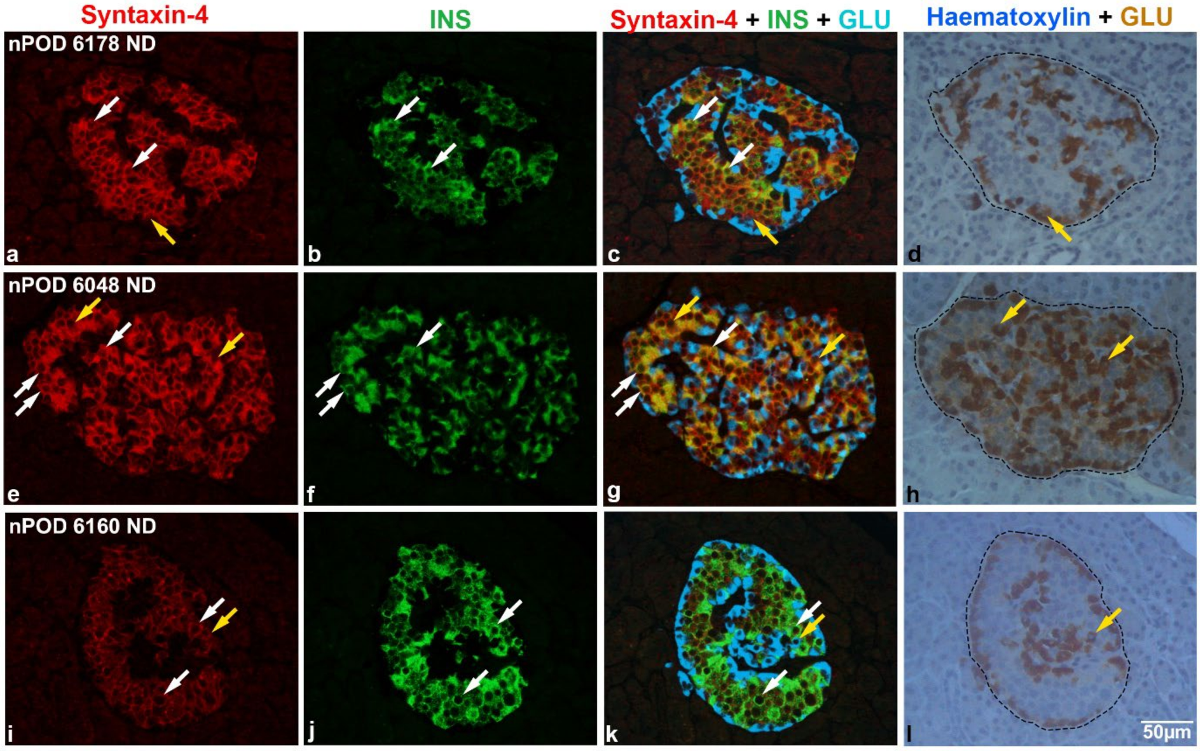

ESM Fig. 4 Immunohistochemical analysis of pancreas sections from selected non-diabetic autoantibody-negative donors showing representative islets triple stained for syntaxin-4 and insulin by immunofluorescence and glucagon by immunoperoxidase. First column: syntaxin-4; second column: insulin; third column shows a merged view of syntaxin-4, insulin and glucagon (following conversion of brown glucagon-positive cells to a cyan colour). The fourth column shows corresponding islets counterstained with haematoxylin where the islet boundaries are indicated by black dashes. White arrows indicate syntaxin-4 in insulin-positive cells; yellow arrows indicate glucagon cells. Scale bar in (l) 50 µm, applies to all micrographs. GLU, glucagon; INS, insulin; ND, non-diabetic; nPOD, Network for Pancreatic Organ Donors with Diabetes

ESM Figure 5

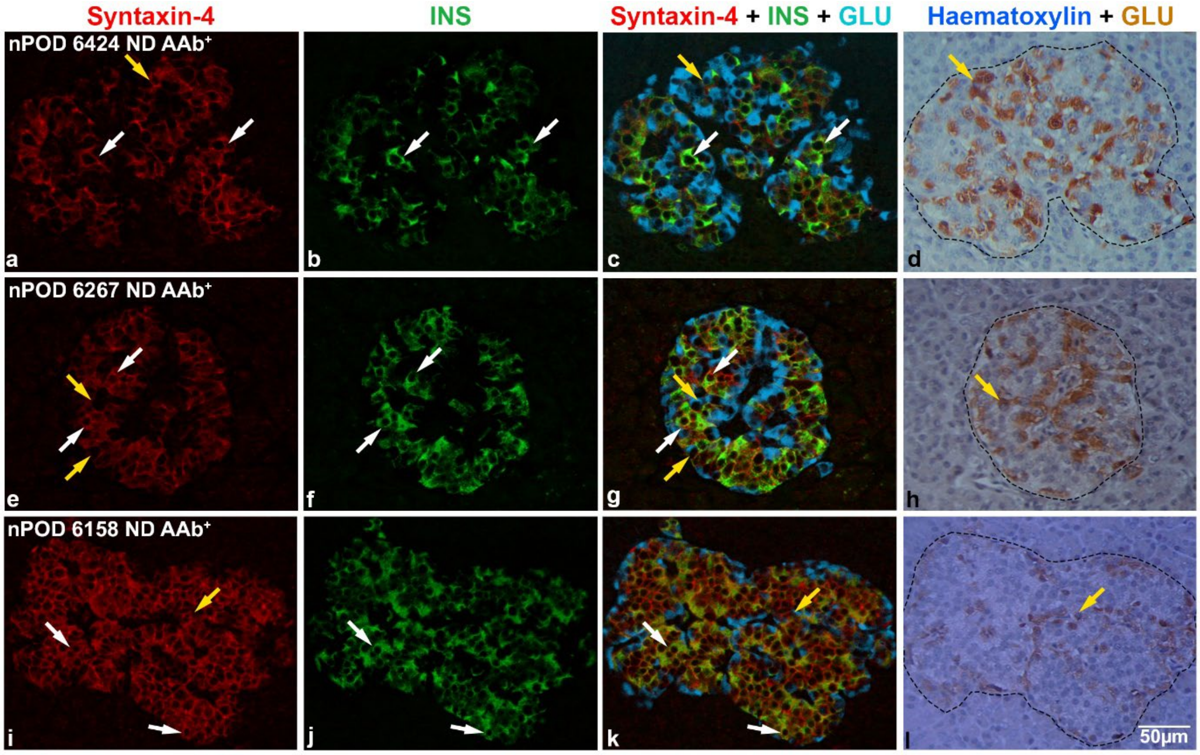

ESM Fig. 5 Immunohistochemical analysis of pancreas sections from selected non-diabetic autoantibody-positive donors showing representative islets triple stained for syntaxin-4 and insulin by immunofluorescence and glucagon by immunoperoxidase. First column: syntaxin-4; second column: insulin; third column shows a merged view of syntaxin-4, insulin and glucagon (following conversion of brown glucagon-positive cells to a cyan colour). The fourth column shows corresponding islets counterstained with haematoxylin where the islet boundaries are indicated by black dashes. White arrows indicate syntaxin-4 in insulin-positive cells; yellow arrows indicate glucagon cells. Scale bar in (l) 50 µm, applies to all micrographs. AAb^+, autoantibody positive; GLU, glucagon; INS, insulin; ND, non-diabetic; nPOD, Network for Pancreatic Organ Donors with Diabetes

ESM Figure 6

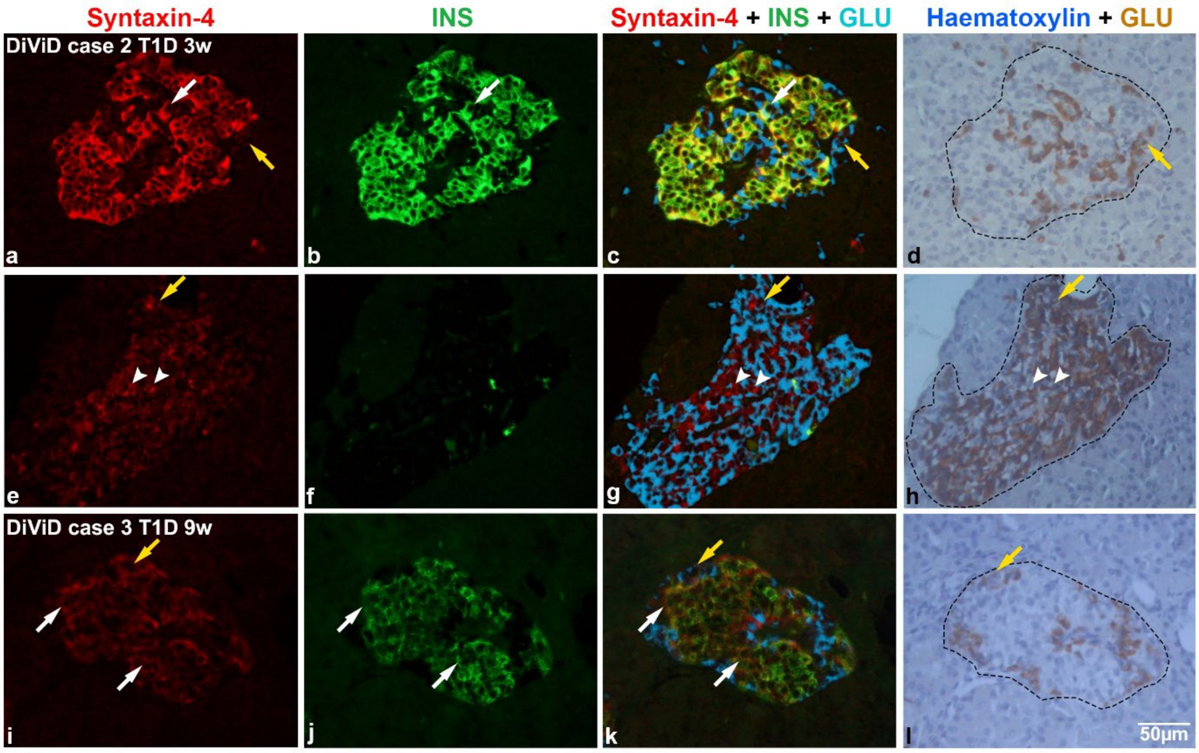

ESM Fig. 6 Immunohistochemical analysis of pancreas sections from newly-diagnosed DiViD cases 2 and 3, showing representative islets triple stained for syntaxin-4 and insulin by immunofluorescence and glucagon by immunoperoxidase. First column: syntaxin-4; second column: insulin; third column shows a merged view of syntaxin-4, insulin and glucagon (following conversion of brown glucagon-positive cells to a cyan colour). The fourth column shows corresponding islets counterstained with haematoxylin where the islet boundaries are indicated by black dashes. White arrows indicate syntaxin-4 in insulin-positive cells; yellow arrows indicate glucagon cells; white arrowheads indicate syntaxin-4 in cells negative for insulin and glucagon. Scale bar in (l) 50 µm, applies to all micrographs. DiViD, Diabetes Virus Detection; GLU, glucagon; INS, insulin; T1D, type 1 diabetes; w, weeks

ESM Figure 7

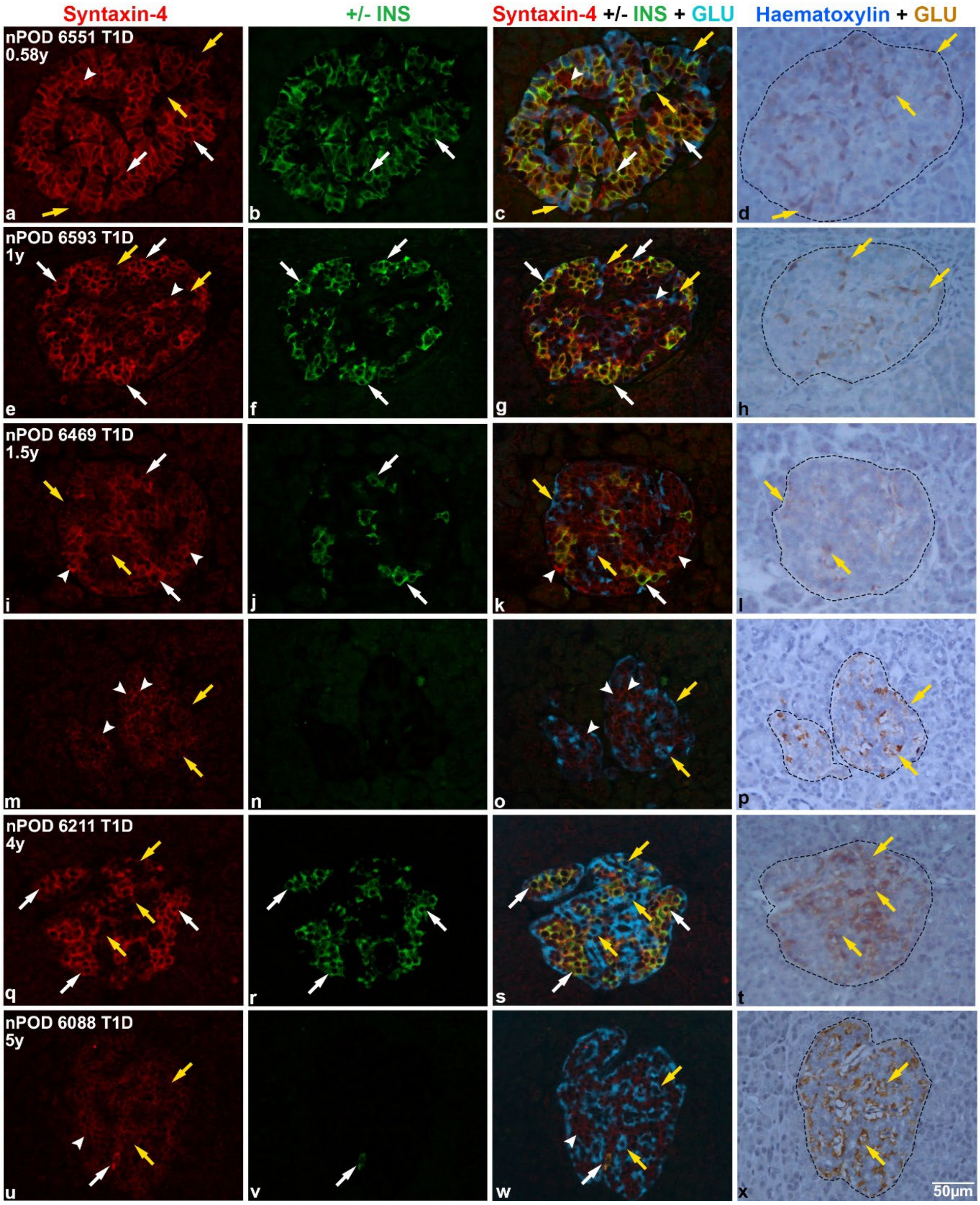

ESM Fig. 7 Immunohistochemical analysis of pancreas sections from long-term T1D donors with diabetes duration from 0.58 year to 5 years, showing representative islets triple stained for syntaxin-4 and insulin by immunofluorescence and glucagon by immunoperoxidase. First

column: syntaxin-4; second column: insulin; third column shows a merged view of syntaxin-4, insulin and glucagon (following conversion of brown glucagon-positive cells to a cyan colour). The fourth column shows corresponding islets counterstained with haematoxylin where the islet boundaries are indicated by black dashes. White arrows indicate syntaxin-4 in insulin-positive cells; yellow arrows indicate glucagon cells; white arrowheads indicate syntaxin-4 in cells negative for insulin and glucagon. Scale bar in (x) 50 μm, applies to all micrographs. GLU, glucagon; INS, insulin, nPOD, Network for Pancreatic Organ Donors with Diabetes; T1D, type 1 diabetes; y, years

ESM Figure 8

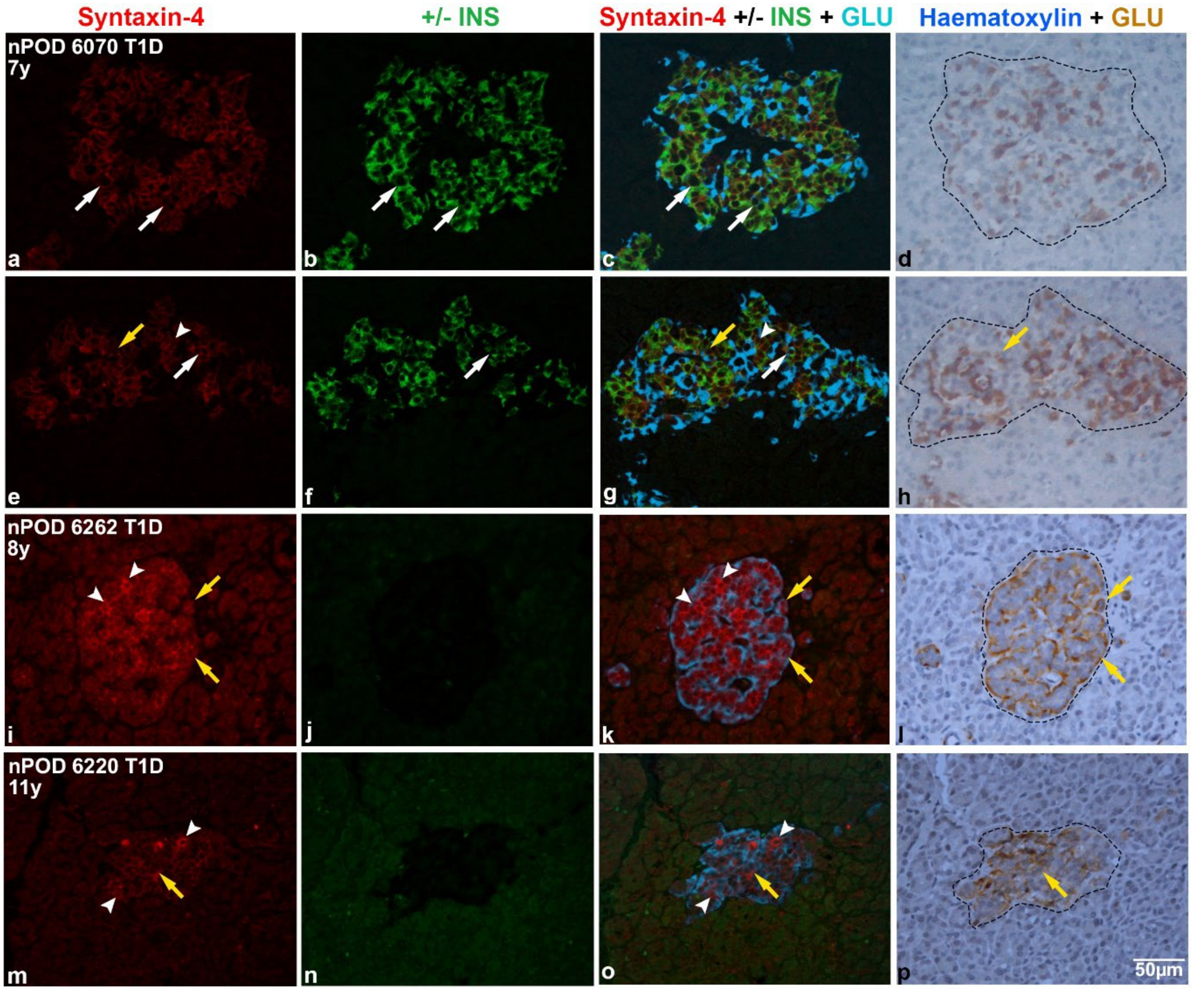

ESM Fig. 8 Immunohistochemical analysis of pancreas sections from long-term T1D donors with a duration of diabetes from 7 years to 11 years, showing representative islets triple stained for syntaxin-4 and insulin by immunofluorescence and glucagon by immunoperoxidase. First column: syntaxin-4; second column: insulin; third column shows a merged view of syntaxin-4, insulin and glucagon (following conversion of brown glucagon-positive cells to a cyan colour). The fourth column shows corresponding islets counterstained with haematoxylin where the islet boundaries are indicated by black dashes. White arrows indicate syntaxin-4 in insulin-positive cells; yellow arrows indicate glucagon cells; white arrowheads indicate syntaxin-4 in cells negative for insulin and glucagon. Scale bar in (p) 50 µm, applies to all micrographs. GLU, glucagon; INS, insulin, nPOD, Network for Pancreatic Organ Donors with Diabetes; T1D, type 1 diabetes; y, years
